# Nanostructured silver dendrites for photon-induced Cysteine dimerization

**DOI:** 10.1038/s41598-019-56517-5

**Published:** 2019-12-27

**Authors:** Chia‐Yu Chang, Yu‐Mei Chen, Yu‐Bin Huang, Chin-Hung Lai, U-Ser Jeng, Ying-Huang Lai

**Affiliations:** 1 Department of Chemistry, Tunghai University, 40704 Taichung, Taiwan; 2 Department of Medical Applied Chemistry, Chung Shan Medical University, Taichung, 40201 Taiwan; 3 National Synchrotron Radiation Research Center, Hsinchu, 30076 Taiwan; 4 Chemical Engineering Department, National Tsing-Hua University, Hsinchu, 30013 Taiwan

**Keywords:** Chemistry, Materials science, Nanoscience and technology

## Abstract

Under a controlled adsorption environment, L-cysteine molecules can be chemically adsorbed to the dendritic silver (Ag-D) surface by electrochemical methods with different functional groups. It is verified by surface-enhanced Raman spectroscopy that under alkaline conditions (pH = 13.50), the two functional groups of thiol and acid are simultaneously adsorbed on the surface of Ag-D, while NH_2_ is far from the surface; under acidic conditions (pH = 1.67), adsorption behavior suggests that both NH_3_^+^ and COO^−^ are oriented toward the Ag-D surface, and that SH is far from the surface. The structure of L-cysteine adsorption under acidic conditions can be further verified by the addition of an L-cysteine molecule through light-induced coupling reaction to form cystine. Finally, *in-situ* two-dimensional Raman scattering spectroscopy confirmed the feasibility and uniformity of the coupling reaction.

## Introduction

L-cysteine is a natural amino acid with a thiol side chain that plays a key role in the organism^1^. For example, abnormal concentrations of cysteine in the blood are important indicators of skin damage and Alzheimer’s disease; the thiol groups of two L-cysteine molecules can form a disulfide-bonded Cys–Cys via oxidation^2,3^. This dimer is called cystine. Its importance lies in (1) its role of stabilizing the spatial structure of the peptide chain, (2) the fact that more there are disulfide bonds, the more stably the protein adapts to the outside, (3) the stable tertiary structure^4^. It is commonly present in keratin and collagen in the hair and skin of the human body, providing structural support or retention of toughness, as well as insulin or oxytocin that regulates physiological functions. In addition, L-cysteine molecules can spontaneously self-assemble with metal surfaces through thiol groups, carboxyl groups, or amine groups, modifying metal properties or increasing the possibility of practical application^5–10^. The modified metal surface can be an important technical application in chemical sensing or catalysis^11–14^. For example, proteins can be more easily and stably adsorbed on the surface to facilitate the provision of a substrate for a nano-process involving biological substances^15,16^. This may help further understand the molecular characteristics of the L-cysteine monolayer and appropriately control the adsorption conditions of the protein; it may even be used to capture and detect heavy metals^17,18^.

Many factors influence the adsorption morphology of L-cysteine on the metal surface, such as temperature, applied potential control, and environmental pH^19^. The selection of the substrate, the molecular environment, the adsorption time, and other conditions are also important factors affecting adsorption and thus the more single and stable adsorption of L-cysteine on the metal. There are many studies on the surface-enhancement Raman scattering (SERS) spectra and theoretical calculations of the adsorption behavior or structure of L-cysteine on the surface of silver nanostructures^4,19–21^. Because of the small Raman scattering cross section of Cysteine molecule and low molecular symmetry, however, the SERS signal of this type of biological small molecule is usually very weak^22^. Only weak enhancement effect occurs: this effect is due to the Herzberg–Teller mechanism (Albrecht’s term B), an enhancement that allows for enhanced non-complete symmetry modes^23–25^. Therefore, high concentrations of target molecules are usually required to generate detectable signals^21^, thus constituting a technical obstacle in the practical application of SERS to biomolecules.

On the surface of the SERS-active substrate, both the physically adsorbed and chemisorbed molecules exhibit an enhanced Raman signal due to the effect of the additive effect^26^. Generally, chemisorbed molecules show an effect more pronounced than that of physically adsorbed molecules^27^. In other words, chemisorbed molecules show the most significant enhancement effects, namely, the highest intensity electromagnetic mechanism (EM) and chemical mechanism (CM) effects^28^. The chemical amplification is affected by the degree of overlap and probability of the bond electron domain between the target molecule and the SERS substrate. However, in SERS measurements, the target molecule can contain a variety of functional groups that can dissociate and subsequently interact with the active substrate. Cysteine molecules contain three different functional groups of amine groups, carboxyl groups and thiol groups^29,30^. Their properties and electrokinetic behavior are largely dependent on the solution pH^31^. Thus, the formation of anionic, zwitterionic, or cationic species is suitable for studying the competitive interaction of functional groups with metal surfaces^32^. In addition, cysteine can form a chemical bond on the metal surface through lone pairs on the functional group. This promotes chemical enhancement through the charge-transfer mechanism, enhancing the Raman signal^33^. However, the cysteine molecule has a small scattering cross section and is not easy to measure, and there are three functional groups that can form a bond with the substrate atom. If the adsorption between the molecule and the substrate is not effectively controlled, the contribution of the chemical amplification effect to Raman scattering makes the signal complex and difficult to judge. In many studies, the adsorption mechanism for this molecular structure is still blurred in SERS analysis. Therefore, this study will examine in detail the adsorption behavior of this molecule on the surface of dendritic silver (Ag-D) on a SERS-active substrate. The cysteine ion state is adjusted by the pH of the solution, and its structure adsorbed on the surface is optimized by a step function. By control of cysteine molecular adsorption behavior, cystine was formed on the surface of SERS substrate by *in situ* SERS measurement and induced coupling reaction of cysteine molecules.

## Results and Discussion

The preparation of high-density and large specific surface area Ag-D deposits on the surface of glassy carbon electrode (GCE) and material characterization are described in SI (Supporting Information). Ag-D is characterized by surface plasmon resonance (SPR), easy preparation, and surface modification. As shown in the Scanning electron microscope (SEM) image of Ag-D in Fig. S1, several branches radially oriented away from the main stem were observed. Numerous secondary leaves were observed on each branch, resulting in the formation of silver dendrites. Thus, the atoms of rich tip, corner, and edge derived from the Ag-D structures were caused by exposure^34^. The dendritic Ag surface showed high Raman enhancement, enabling the detection of analytes from dilute solution by SERS. 4-Nitrothiophenol was used as the probe molecule to examine the analyte-concentrating ability and SERS activity of Ag-D. Quantitative SERS detection using the Ag-D has detection limits in the nanomolar (5 nM) concentration range^35^. It is a very good substrate for SERS. However, surface silver atoms are reactive and easily oxidized, and preservation and SERS measurement conditions require precise control. In this study, we used L-cysteine as the target molecule, and the Ag-D electrode was a surface-enhanced Raman substrate; that is, the reactivity of the surface of the SERS substrate to the target molecule was investigated. In aqueous solution, the functional groups of L-cysteine ionize with different net charges as the pH changes (Fig. S3), with pKa values of 1.9 (carboxyl), 8.4 (thiol), and 10.5 (amine)^36^. By controlling the pH value of the solution, L-cysteine changes the charge of the functional group under different acid–base conditions, and there are chemical adsorption behaviors and structure changes on Ag-D surface. In addition, the Henderson–Hasselbalch equation can be used to estimate the ratio of conjugate acid/base pairs. In the electrochemical system, we used the step function mode to control the potential, so that L-cysteine continues to adsorb onto and desorb from the surface of Ag-D, thus achieving the most stable and uniform adsorption structure, as well as even distribution on the silver surface.

**Figure 1:**
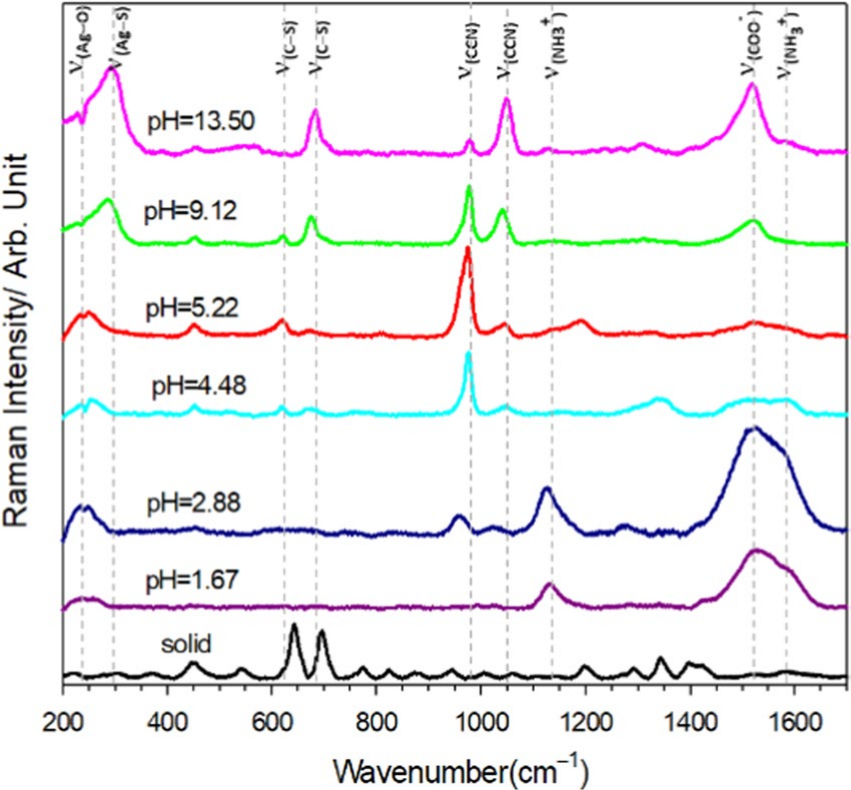
SERS spectra of 100 μM L-cysteine as a function of pH on Ag-D electrodes. The solid black line is the Raman scattering spectrum of the solid L-cysteine. The pH is indicated in the figure. The experimental measurement conditions of the SERS spectrum were 1 second, average of five spectra, incident light wavelength of 532 nm, and power density of 8.0 mW at the sample position.

**Figure 2:**
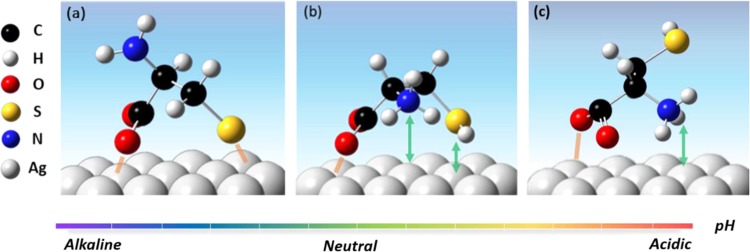
Schematic diagram of L-cysteine adsorption on the silver surface: structure A (**a**), structure B (**b**) and structure C (**c**).

Figure 1 shows the SERS spectrum of 100 μM L-cysteine as a function of pH on an Ag-D electrode. First, when pH = 13.50, characteristic peaks of 288, 679, and 1519 cm^−1^ can be observed. They are for ν(Ag–S), ν(C–S), and ν(COO^−^) respectively, as shown in Table S1 ^32,37,38^. It can be seen that L-cysteine directly bonded to the silver surface by S^−^, and that C–S bond has a clear signal enhancement and a relatively low wavenumber compared with the L-cysteine solid powder. It is speculated that in this environment, the sulfur generates a strong interaction (chemical bond) between the negative charge of the deprotonation of thiol group and positive side of the silver surface, and the increase in the COO^−^ signal intensity indicates that the COO^−^ and the silver surface have a strong interaction. It can be seen that this part can also be verified by the ν(Ag–O) shoulder at 247 cm^−1^. From this result, it is inferred that the adsorption behavior of L-cysteine is that COO^−^ and S^−^ adsorb on the surface of Ag-D, while NH_2_ is far from the surface, as shown in Fig. 2(a) structure A. At pH = 5.22 or 4.48, close to the isoelectric point of L-cysteine, the characteristic peak is 247 cm^−1^, and its vibration corresponds to ν(Ag–O), indicating that COO^−^ forms a bond with the silver surface. The characteristic peak is ν(C–S) at 674 cm^−1^ shifts to 620 cm^−1^. As the pH value is lower, S gradually changes to the form of SH. No bond formed at this time, but it was still close to the surface of the Ag-D, so that the increase in this signal was observable. Therefore, it is inferred that the adsorption behavior is such that COO^−^ and SH are oriented toward the Ag-D surface, and that NH_3_^+^ is relatively far from the surface, as shown in Fig. 2(b) structure B. When the pH value reaches the acidity, pH = 1.67, ν(Ag–O) at 247 cm^−1^ and ν(COO^−^) at 1525 cm^−1^ increased, indicating that COO^−^ and the dendritic silver surface bonded. In addition, there is a tendency for the shoulder peak at 1586 cm^−1^ to appear next to 1525 cm^−1^. It is presumed that the signal is enhanced with the vibration ν(NH_3_^+^) corresponding to 1127 cm^−1^, indicating that NH_3_^+^ is also close to the surface of Ag-D. Finally, it was observed that there was no characteristic peak of ν(C–S) between 600 and 750 cm^−1^, indicating that SH was far from the surface. Therefore, this adsorption behavior suggests that both NH_3_^+^ and COO^−^ are oriented toward the Ag-D surface, and that SH is far from the surface, as shown in Fig. 2(c) structure C. Among them, between pH = 13.50 to 5.22 and pH = 4.48 to 1.67, respectively, is a transition state, and there may be two different forms of L-cysteine. Therefore, characteristic peaks can be observed for ν(C–S) at 600–680 cm^−1^ and for ν(C–N) at 950–1050 cm^−1^. When the pH is 9.12, there are two structures, as shown in Fig. 2(a,b); at pH 2.88, there are two structures, as shown in Fig. 2(b,c). With the change in pH, we could observe the relative intensity, and the ν(C–S) characteristic peak of 674 cm^−1^ gradually shifts to 620 cm^−1^. At this time, the torsion angle of L-cysteine χ_1_ (N–C_α_–C_β_–S) is changed from trans to gauche. However, the ν(C–N) characteristic peak at 1024 cm^−1^ is also gradually shifted to low frequency to 973 cm^−1^. This phenomenon is presumed to be due to changes in adsorption behavior and structure, which in turn causes changes in the electron density of C–S or C–N and causes the change in Raman scattering signals.

In different acid–base environments, cysteine forms a chemical bond with the silver atoms on the surface of the dendritic silver SERS-active substrate. In addition to the electromagnetic field enhancement considering the SPR effect of dendritic silver, there is a clear chemical contribution. In addition, L-cysteine can be selectively adsorbed onto the surface of the Ag-D electrode by controlling the pH and potential. Thus, L-cysteine can be further used as a finishing layer for developing subsequent, broader applications by reaction with different exposed functional groups with additional added compounds.

**Figure 3:**
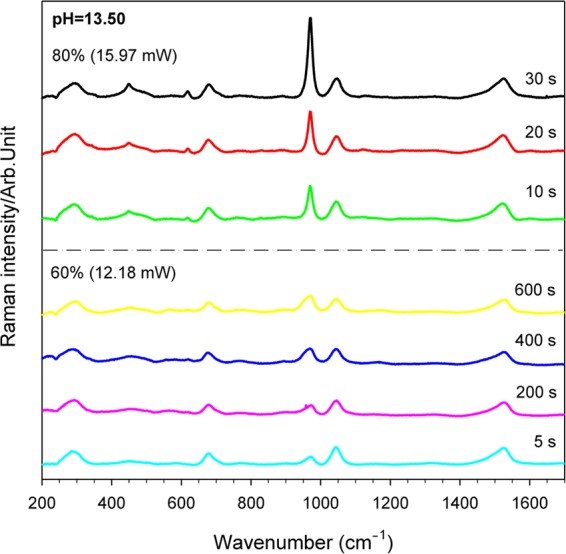
The SERS spectra of 100 μM L-cysteine on the Ag-D electrode with light intensity and time at pH = 13.50 and laser wavelength of 532 nm. The laser light intensity and the exposure time are shown in the figure.

The effects of laser intensity and exposure time on the adsorption structure of L-cysteine were further investigated (Fig. 3). The SERS spectrum of L-cysteine adsorbed on the Ag-D surface with different light intensities and irradiation time at pH = 13.50 is shown. The signal characteristic peak corresponding to the wavenumber can be seen in Table S1. The time was 5 seconds/time for immediate response measurement. When the laser power is 60% (12.18 mW) for the first 5 seconds, the ν(C–N) signal intensity of 1042 cm^−1^ is stronger than that of ν(C–N) 972 cm^−1^; only at 680 cm^−1^, is the ν(C–N) signal intensity consistent with the result for pH = 13.50 in Fig. 1. However, when the exposure time was increased to 400 seconds or even 600 seconds, it was observed that the characteristic peak of 972 cm^−1^ was gradually stronger than that of ν(C–N) at 1042 cm^−1^. This means that under the same illumination intensity, prolonged exposure causes L-cysteine to slowly undergo partial structure reorganization. When the laser power is increased to 80% (15.97 mW), it is clear that after 10 seconds of exposure, not only was the characteristic peak of 972 cm^−1^ significantly enhanced, but also the ν(C–S) of 680 cm^−1^ had a trend of signal enhancement. When the exposure time reached 30 seconds, the intensity of the two characteristic peaks became stronger. Increasing the light intensity can make L-cysteine result in faster structure variation, further changing the electron distribution of C–S and C–N and resulting in this growth and decline. The SERS spectrum with pH has been judged to have an adsorption behavior at pH of 13.50, as shown as structure A. In the literature^32,39^, the NH_2_, COO^−^ and S^−^ groups in the three structures of L-cysteine are close to the silver surface, and the torsion angle of χ_1_(N–C_α_–C_β_–S) is the most stable state when it is structure B. Therefore, the inference is due to the fact that L-cysteine itself has a rotating dihedral angle. When the laser source supplied additional energy to L-cysteine, driving the original structure A, it was easier to change to a state structure B, which is adsorbed on the Ag-D surface. On the other hand, L-cysteine modified with pH = 5.22 was further exposed to 60% (12.18 mW) of the laser power, as shown in Fig. S4. From the observation of the SERS spectrum with time, it was found that the structure variation of L-cysteine chemisorption was not obvious. This means that providing energy to the most stable adsorption state under the same conditions does not further change the adsorption structure of L-cysteine, and that only the Raman scattering intensity decreases overall. It is apparent that the adsorbed molecule still maintains structure B.

It has been confirmed that the adsorption configuration of L-cysteine on the surface of the Ag-D electrode can be successfully controlled by the solution pH environment and the adsorption–desorption potential. Next, the functional groups of the chemisorbed modified layer will be further utilized to form a dimer with a laser-light-induced dimerization reaction with an added L-cysteine molecule. Upon oxidation of the two L-cysteine monomers, a cystine with a disulfide bond is formed. From the experimental results (Fig. 1) it is known that three adsorption forms can be obtained with the change in acid–base environment, which are located at pH 1.67, 5.22, and 13.50. Therefore, the following experiments will use the L-cysteine controlled by these three pH values as the intermediate layer, and add L-cysteine aqueous solution to carry out laser-induced reaction to form cystine. They will explore the impact of the intermediate layer dimerization of different pH values on the reaction.

**Figure 4:**
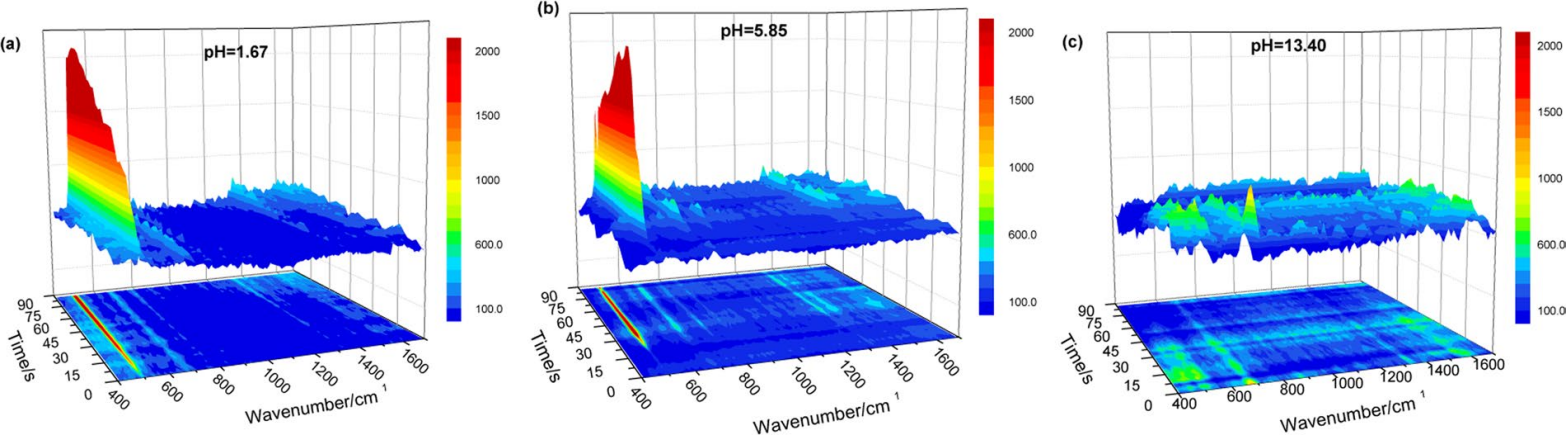
SERS spectra of the reaction time changes corresponding to cystine formed by light on the surface of Ag-D after L-cysteine modification, when a drop of 0.01 M L-cysteine in water was added at (**a**) pH = 1.67, (**b**) pH = 5.85, and (**c**) pH = 13.40.

The excitation wavelength was 532 nm, the objective lens was 50 times, and the integral time of each spectrum was 3 seconds/time for real-time reaction measurement. Figure 4(a) shows the SERS spectrum of the cystine reaction time on the surface of silver formed by L-cysteine at pH = 1.67. In the first 3 seconds of the reaction, there was no ν(S–S) at 500 cm^−1^ in the SERS spectrum, and no dimerization occurred at this time. The addition of a drop of 0.01 M L-cysteine aqueous solution onto the surface of the substrate will wet the surface and change the original chemical environment, so that the original signal disappears. When the reaction time was 6 seconds, it was observed that ν(S–S) gradually appeared, indicating that the thiol groups of the two L-cysteine monomers began to dimerize to form cystine^37^. After 150 seconds of continuous reaction, the intensity of the ν(S–S) signal peak had reached near saturation, and the relatively weak characteristic peaks of other signals were obvious; 602, 664, 1330, and 1401 cm^−1^ correspond to vibrations of ν(C–S), ν(C–S), δ(NC–H), and ν(COO^−^), respectively.

Figure 4(b) is a SERS spectrum of L-cysteine forming cystine during reaction on the silver surface at pH = 5.85. In the first 12 seconds of the reaction, no dimer was formed. When the reaction time was 15 seconds, we could observe that the ν(S–S) signal peaks at 500 cm^−1^ gradually appeared, and the other signal peaks increased with time. When the reaction reaches 90 seconds, the signal peak intensity tends to be saturated, indicating that the cystine almost completely formed at this time. Comparing the reaction times for pH = 1.67 and 5.85, we found that the reaction time of the latter was longer than that of the former. It is speculated that at lower pH values, the presence of L-cysteine is relatively simple. As shown in Fig. 2(c), the thiol group is mainly away from the silver surface; thus it is easier and faster to form a dimer. The latter is close to the isoelectric point. Under this condition, other forms exist and the thiol group is relatively close to the silver surface, making the reaction less likely to occur.

Finally, Fig. 4(c) shows the SERS spectrum of L-cysteine forming a cystine in reaction with time, with change on the silver surface at pH = 13.40. It can be observed that at reaction time of 90 seconds, ν(S–S) of 500 cm^−1^ still does not appear. This means that under these conditions, cystine cannot be formed by dimerization reaction. However, we observed a slight signal peak; we presume that unreacted L-cysteine monomer molecules randomly located on the surface of the L-cysteine-modified Ag-D substrate. From the experimental results of the pH control, it is known that in this pH environment, the sulfur end of L-cysteine bond with the silver surface, and that there is no exposed thiol group; thus, cystine does not form.

**Figure 5:**
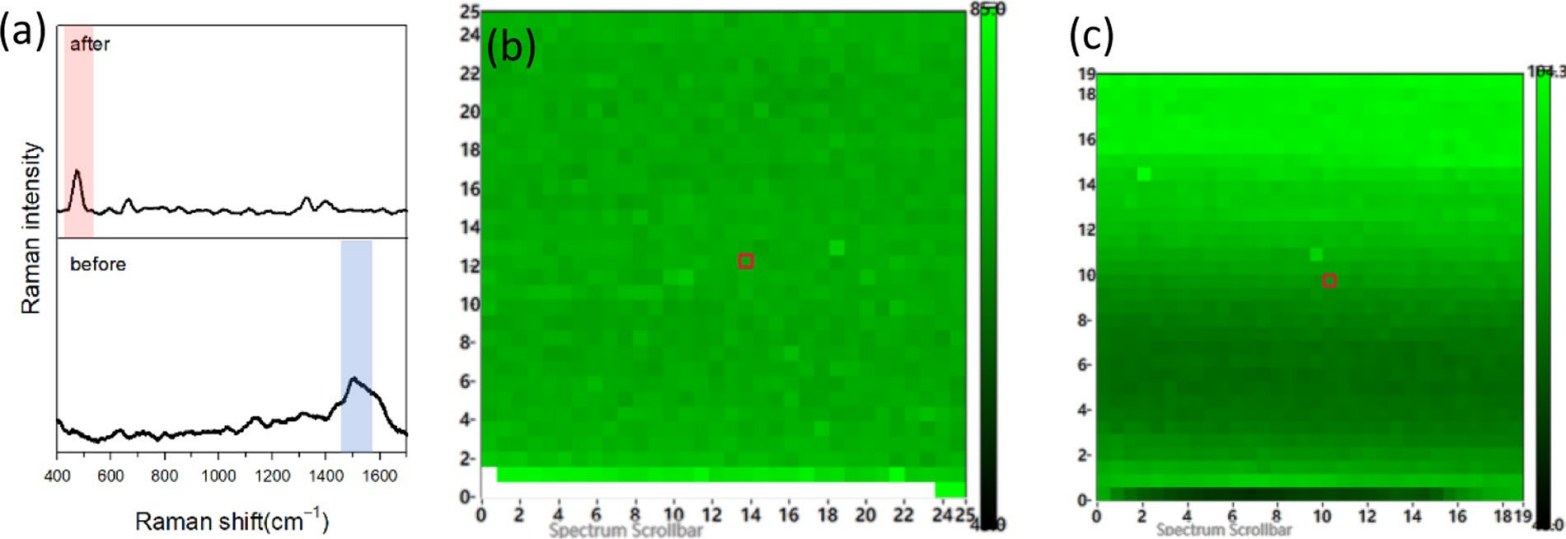
SERS mapping of a drop-casting Cysteine on cysteine-modified Ag-D. (**a**) The SERS spectra of cysteine before and after exposure coupling, (**b**) high-resolution Raman integral intensity maps of 1500 cm^−1^ for cysteine(25 μm × 25 μm), and (**c**) 500 cm^−1^ Raman bands for cystine(19 μm × 19 μm).

It was confirmed that the L-cysteine coupling reaction occurred at a single point. The uniformity of the reaction on the surface of the Ag-D after L-cysteine modification was further investigated. First, the Raman spectral measurement range of 25 μm × 25 μm (32 × 32 pixel) was mapped by using an optical microscopy image. The SERS spectra of this range were scanned to match the results of L-cysteine modification on Ag-D at pH = 1.67 (Fig. 5(a)). According to the two-dimensional image of the signal intensity at 1500 cm^−1^ corresponding to the ν(COO^−^) vibration of L-cysteine (Fig. 5(b)), the intensity distribution of the difference is about ±3% within this measurement range. The electrochemically modified L-cysteine has good uniformity. Next, a drop of 0.01 M L-cysteine solution was added to measure the same region. The spectrum in Fig. 5(a) also met the SERS spectrum of cystine after exposure coupling. Raman mapping was established by ν(S–S) vibration at a signal intensity of 500 cm^−1^ (see Fig. 5(c)). After the exposure reaction, the intensity variation of the two-dimensional spectrum of ν(S–S) is about ±17%, and the reaction uniformity is very good. From this, it can be determined that each pixel in the range of 19 μm × 19 μm (32 × 32) has the generation of S–S. According to Raman mapping verification, under acidic conditions (pH = 1.67), L-cysteine chemical adsorption on the surface of Ag-D can further lead to reaction with free L-cysteine to form cystine. The uniform area of modification and reaction is ≥19 μm × 19 μm.

## Conclusion

After a series of SEM, XRD, and XPS characterization, it was proved that the high-density Ag-D electrode can be prepared by electrochemical deposition method. It is suitable for surface-enhanced Raman substrate, and it can successfully utilize the electrochemical method of adsorption of L-cysteine on it.

The structure of the adsorbed molecule L-cysteine can be optimized by adjusting the adsorption and desorption of the target molecule on the Ag-D surface multiple times by electrochemical potential. In the SERS spectrum of the effect of pH on the adsorption of L-cysteine, three different adsorption behaviors can be obtained by adjusting the pH. This is the action of COO^−^ and S^−^ with the silver surface, and NH_2_ movement away from the surface (alkali); or with NH_3_^+^ and COO^−^ reacts on the silver surface, while the SH moves away from the surface (acid). The last one is when NH_3_^+^, COO^−^, and SH are all close to the silver surface. Therefore, it is possible to simultaneously adjust the environmental pH value and to control the adsorption and desorption potential, so that L-cysteine has different adsorption structures. However, in the SERS spectrum that changes the laser power and illumination time, L-cysteine is converted from the structure A (trans structure) to the more stable structure B (gauche) because of the applied energy (pH = 13.40).

Finally, the three adsorption states obtained under the control of pH were respectively subjected to oxidation reaction by light-induced L-cysteine. It was confirmed that the pH value at the most acidic and isoelectric point conditions, allows two L-cysteines to undergo oxidation reaction to form a disulfide because of the non-bonded thiol group. However, as the pH is higher, the reaction rate is slower and less likely to proceed. Two-dimensional Raman scattering spectroscopy confirmed the feasibility and uniformity of the coupling reaction. The modified cysteine on the dendritic silver electrode has the possibility of further react in the measurement range. It can be seen that in this study, we can effectively control the adsorption behavior of L-cysteine and that we can used it as a modification to improve the surface selectivity and to conduct more chemical reactions.

### Experimental method and material property identification

#### Preparation of nanostructured silver dendrites (Ag-D)

Ag-Ds were prepared through a modification of our previous report^35^. The glassy carbon electrode (12 mm × 5 mm × 1 mm) was polished with of 0.3 μm and 0.05 μm alumina powder, and then ultrasonically shaken with ethanol and deionized water for 15 minutes. Finally, the glassy carbon electrode was rinsed with deionized water. The dendritic silver was directly deposited on the glassy carbon electrode and was easily peeled off. Therefore, before preparing the dendritic silver, nanogold was deposited on the surface of the glassy carbon electrode for 500 seconds, and then dendritic silver was deposited thereon. About 30.0 mL of 1.0 mM tetrachloroauric acid, 0.10 mM cysteine, and 0.50 M aqueous sulfuric acid solution was prepared. A three-electrode electrochemical system (CHI6111) was used. The reference electrode was saturated calomel electrode (SCE), the auxiliary electrode was platinum wire, and the working electrode was glassy carbon. We used the step function for gold electrodeposition for 500 seconds. The parameter settings were 0 to −0.8 V potential range, 0.1 s step time, and 5000 step segments. Next, dendritic silver deposition was performed. The solution was changed to 1.0 mM silver sulfate, 0.10 mM cysteine, and 0.50 M aqueous sulfuric acid solution, and we used about 30.0 mL. Three electrodes were reference electrodes: silver wire, auxiliary electrode (platinum wire), and working electrode (gold-modified glassy carbon electrode). We used the step function for silver electrodeposition for 1000 seconds. Parameter settings were −0.2 to −1.2 V potential range, 0.1 s step time, and 10000 step segment. The deposited dendritic silver electrode was taken out, washed with deionized water, and dried with nitrogen. The dendritic silver electrode should be washed with 0.2 M Na_2_S_2_O_3_ for 10 seconds to remove the surface Ag_2_SO_4_ residue to obtain a Raman substrate with no background interference signal. The dendritic silver obtained was identified by SEM, XRD, and XPS.

#### Preparation of raman samples

First we prepared a 100 μM L-cysteine solution and adjusted the pH to be measured with KOH and HNO_3_ at approximately 30 mL. We set up a three-electrode system: the reference electrode was silver wire, the auxiliary electrode was platinum wire, and the working electrode was dendritic silver electrode. We used a step function to adsorb L-cysteine for 1500 seconds. Parameter settings were potential range of −0.2 to −1.2 V, step time of 0.1 s, and step segment of 15000. The L-cysteine-modified dendritic silver electrode was taken out, rinsed, dried with N_2_ gas, and then measured. Samples were irradiated with a laser illumination of 532 (25 mW), focused on a sample of 1 μm diameter with a 50X objective lens (Plan N, Olympus), MRI532S, Protrustech Co., Ltd, Taiwan.

#### *In situ* raman spectra

All Raman scattering spectra were obtained at 532 nm excitation wavelength was corrected by the signal peak of polystyrene at 1001.4 cm^−1^. The objective lens magnification was 50 times. The integration time was as indicated in the figure, and the instantaneous reaction measurement was performed. A drop of 0.01 M L-cysteine aqueous solution was placed onto the surface of the L-cysteine-modified dendritic silver obtained under the conditions of pH = 1.67, 5.85, 13.40. The coupling reaction was induced by a 532 nm light source while the change in the SERS spectrum was monitored during the reaction. The source intensity was 9.42 mW at the sample position.

## Supplementary information

Supplementary Information
