# Supplementary material for: Nanostructured silver dendrites for photon-induced Cysteine dimerization
Source: Sci Rep. 2019 Dec 27;9:20174. doi: 10.1038/s41598-019-56517-5 (PMC6934660; doi:10.1038/s41598-019-56517-5)
Supplement: Supplementary file 1 — Supplementary Information [file 41598_2019_56517_MOESM1_ESM.docx]

**Supporting information**

**Nanostructured silver dendrites for photon-induced Cysteine dimerization**

Chia‐Yu Chang,^a^ Yu‐Mei Chen,^a^ Yu‐Bin Huang,^a^ Chin-Hung Lai,^b^ U-Ser Jeng,^c,d^ Ying-Huang Lai^a,*^

^a^ Department of Chemistry, Tunghai University, 40704, Taichung, Taiwan

^b^ Department of Medical Applied Chemistry, Chung Shan Medical University, Taichung 40201, Taiwan

^c^ National Synchrotron Radiation Research Center, Hsinchu 30076, Taiwan.

^d^ Chemical Engineering Department, National Tsing-Hua University, Hsinchu, 30013, Taiwan

**Corresponding author**

E-mail: yhlai@go.thu.edu.tw

**Ag-D property identification**

**1. Scanning electron microscope (SEM)**

From the SEM image of Figure S1. (a), uniformity was observed at 6000 times. Figure S1. (b) is at 30000 magnification; here, the main features of the tree structure can be observed. The trunk is approximately 2 μm and the stem has many tips. Because of its high density and fine structure, it provides an excellent Raman effect on measurement. In the prior Ag-D preparation method, it is necessary to prewash with Na_2_S_2_O_3_ before the application.^1^ Since the precursor of the Ag-D electrode is Ag_2_SO_4_, the solubility product (*K*sp = 1.4 × 10^−5^) is for a poorly soluble electrolyte. Therefore, during the deposition of silver atoms, Ag_2_SO_4_ remains on the surface, interfering with the Raman signal. By using Na_2_S_2_O_3_ and residual Ag_2_SO_4_ mis-synchronization and exchange to form a more soluble Ag[S_2_O_3_]_n_, we can remove the residual Ag_2_SO_4_ on the surface. In order to ensure that the structural integrity after the bubble washing is not destroyed, we compare the images before and after the bubble washing. Figure S1 (c), obtained at 6000 times, shows the tree structure, which can be roughly evenly distributed. Figure S1 (d) is obtained at 30,000 times, shows the tree structure integrity and the detailed structure.

**F
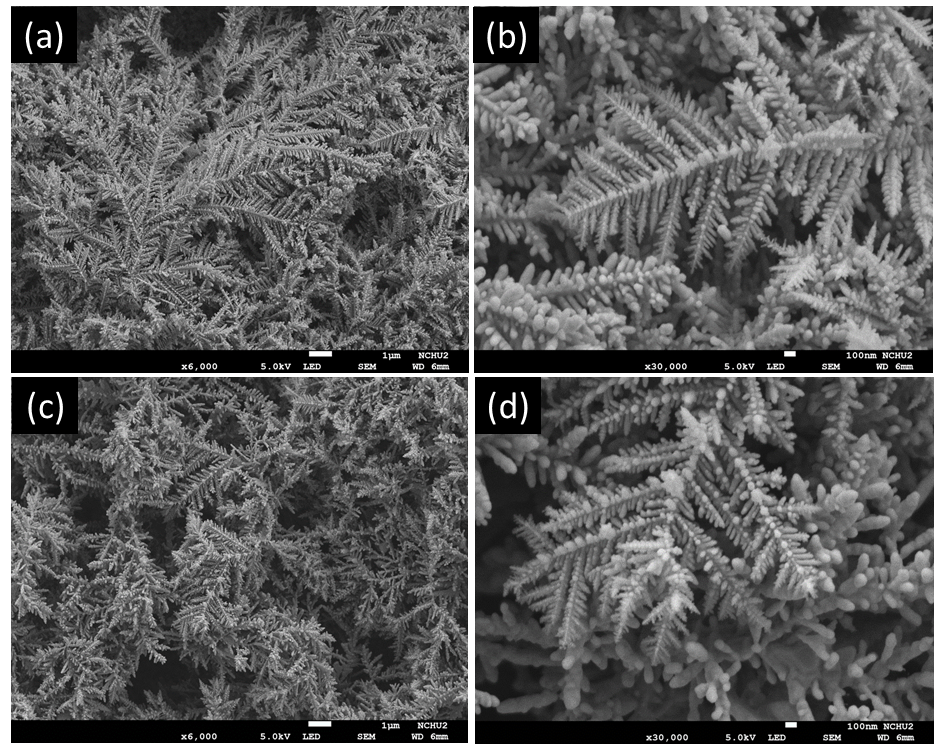
igure S1.** (a), (b) Ag-D before soaking on glassy carbon with Na_2_S_2_O_3_ (c) and (d) SEM image after soaking with Na_2_S_2_O_3_ for 10 seconds

**2. X-ray diffraction (XRD)**

The dendritic silver electrode prepared by pulsed electrochemical deposition, which was used to further study its crystal structure (Figure S2. (a)). The five diffraction signal peaks of (a) are at 38.18°, 44.39°, 64.57°, 77.55°, and 81.72°, and the corresponding crystal faces are (111), (200), (220), (311), and (222), respectively.^2^ It is confirmed that the structure is crystalline silver of the FCC stack structure of the vertical aspect, and the diffraction signal peak is relatively thin, indicating that the dendritic silver structure is highly crystalline.

**3. X-ray photoelectron spectroscopy (XPS)**

Figure S2. (b) shows a dendritic silver electrode prepared by pulsed electrochemical deposition, with surface element analysis by XPS. This experimental measurement of the light source (hν = 620 eV) detailed scanning of the silver 3d orbital domain. The binding energies of silver are 3d_5/2_ = 368.3 eV and 3d_3/2_ = 374.3 eV, which are consistent with the binding energy of reduced silver.

**
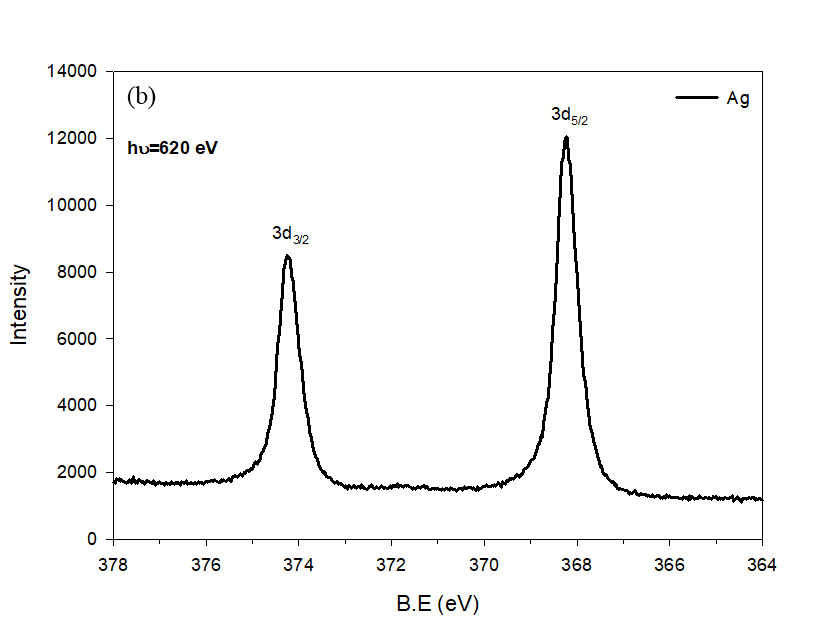
**

**Figure S2.** (a) XRD and (b) XPS diagram of Ag-D on glassy carbon electrode

**Figure S**
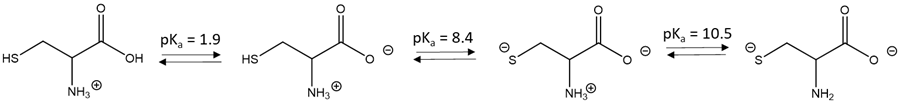
**3**. *K_a_* value change of L-cysteine

Table S1. Corresponding vibrational shape of the characteristic peak of the SERS spectrum of L-cysteine
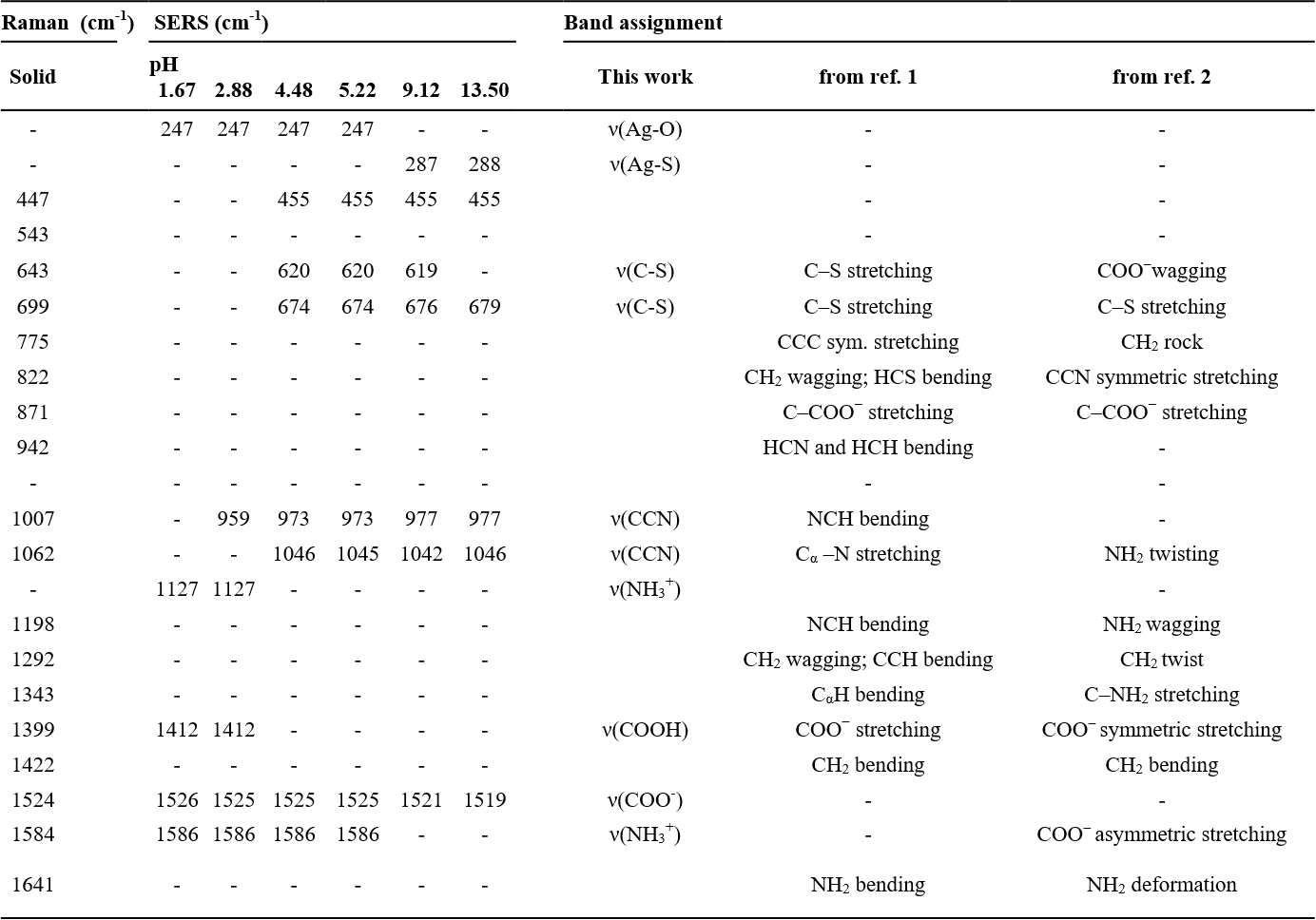


ref.1 Diaz Fleming, G.; Finnerty, J. J.; Campos-Vallette, M.; Célis, F.; Aliaga, A. E.; Fredes, C.; Koch, R., Experimental and theoretical Raman and surface-enhanced Raman scattering study of cysteine. *Journal of Raman Spectroscopy* **2009,** *40* (6), 632-638.

ref.2 Podstawka, E.; Ozaki, Y.; Proniewicz, L. M., Part I: Surface-Enhanced Raman Spectroscopy Investigation of Amino Acids and Their Homodipeptides Adsorbed on Colloidal Silver. *Applied Spectroscopy* **2004,** *58* (5), 570-580.

**Figure S4.** SERS spectra of 100 μM L-cysteine on Ag-D electrode with light intensity and time at pH = 5.22. The laser wavelength is 532 nm. The laser light intensity and exposure time are shown in the figure.

Ref.

1 Teng, Y., Liu, W., Lan, M., Ma, S. & Ma, C. Porous and Dendritic Structure of Screen-Printed Electrode for in-situ Electrochemical Surface-Enhanced Raman Scattering. *Analytical Letters* **49**, 299-306, doi:10.1080/00032719.2015.1070164 (2016).

2 Cho, F.-H., Kuo, S.-C. & Lai, Y.-H. Surface-plasmon-induced azo coupling reaction between nitro compounds on dendritic silver monitored by surface-enhanced Raman spectroscopy. *RSC Advances* **7**, 10259-10265, doi:10.1039/C7RA00374A (2017).
